# THE COGNITIVE DECLINE TOWARD DEMENTIA AMONG NURSING HOME RESIDENTS

**DOI:** 10.1093/geroni/igae098.3002

**Published:** 2024-12-31

**Authors:** Audai Hayajneh

**Affiliations:** Jordan University of Science and Technology, Irbid, Irbid, Jordan

## Abstract

A scarcity of studies have examined the factors associated with mild cognitive impairment (MCI) as well as moderate cognitive impairment-as considered as dementia- among nursing home residents (NHRs). The aim of this study was to recognize the nature of relationship between the health risk variables, such as comorbidities, length of stay at hospital, the quality of life, the occurrence of depression, disability, and frailty, and dementia in NHRs. The study deployed cross-sectional design, using a convenient sample of 182 nursing home residents. The settings of the study were in the middle region of Jordan. Both stages of the MCI and dementia were determined through Montreal cognitive assessment (MoCA). Bivariate and multivariate analyses were utilized to explore the relationship between the health risk variables, and each of the MCI and dementia. There were significant differences between the stages of cognitive impairment (The MCI and dementia) in terms of age, the quality of life, comorbidities, depression, frailty. The variation in mental ability scores was dependent on the marital status of the participants, the monthly income, recently hospitalized, the level of depression, and frailty among older adults residing at nursing homes. The results of this study contribute significantly to building a healthcare management protocol to deal with nursing home residents with depressive symptoms as well as the deferral of the trajectory of development of dementia among those risky residents.

